# Adipokine levels and carbohydrate metabolism in patients diagnosed de novo with polycystic ovary syndrome

**DOI:** 10.5339/qmj.2021.34

**Published:** 2021-08-31

**Authors:** Ewelina Kolan′ska-Dams, Joanna Boinska, Maciej W. Socha

**Affiliations:** ^1^Department of Pathophysiology, Faculty of Pharmacy, Collegium Medicum in Bydgoszcz, Nicolaus Copernicus University In Torun′, Poland E-mail: joanna_boinska@cm.umk.pl; ^2^Department of Obstetrics, Gynecology and Gynecological Oncology, Faculty of Medicine, Collegium Medicum in Bydgoszcz, Nicolaus Copernicus University, Poland

**Keywords:** polycystic ovary syndrom, apelin, leptin, carbohydrate metabolism

## Abstract

Introduction: Central obesity appears to play a major role in the pathogenesis of metabolic disorders in polycystic ovary syndrome. Insulin resistance and carbohydrate disorders are associated with dysfunctional secretion of various adipokines by the adipose tissue.

Objectives: This study aimed to evaluate leptin, apelin, and visfatin against a background of carbohydrate metabolism parameters in patients diagnosed *de novo* with polycystic ovary syndrome (PCOS).

Material and methods: The study group consisted of 40 patients with PCOS (mean age, 29 years) diagnosed in accordance with the American Society for Reproductive Medicine criteria from 2003. The control group consisted of 37 clinically healthy women (mean age, 26 years). All controls had regular menses and no clinical or biochemical signs of hyperandrogenism. Concentrations of leptin, apelin, visfatin, and insulin were measured by immunoenzymatic methods. Glucose concentrations were determined using spectrophotometry.

Results: Significantly higher concentrations of leptin, insulin, homeostatic model assessment for insulin resistance (HOMA-IR) index, and the immunoreactive insulin (IRI)/glucose index were found in the PCOS group than in the control group. Notably, the concentration of apelin was over five times lower in the PCOS group than in the control group. In patients with PCOS, a positive correlation was found between the concentrations of insulin and leptin and concentrations of leptin and IRI/glucose. Patients of the PCOS group with body mass index (BMI) ≥  25 had significantly higher values of leptin, insulin, HOMA-IR index, and IRI/glucose index than patients of the PCOS group with normal BMI. In the PCOS group, a positive correlation was found between BMI and leptin concentration (r = 0.7176; *p* < 0.0001) and carbohydrate metabolism, such as insulin (r = 0.5524; p = 0.0003), glucose (r = 0.3843; p = 0.0157), HOMA-IR (r = 0.5895; p < 0.0001), and IRI/glucose (r = 0.3872; p = 0.0163). These findings were not observed in the control group.

Conclusions: (1) Increased leptin concentration observed in women diagnosed *de novo* with PCOS as well as positive correlations between leptin and HOMA-IR, and IRI/glucose and BMI may indicate a potential role of leptin in the reduction of tissue sensitivity to insulin. (2) Significantly lower apelin concentration in the PCOS group (>5 fold) than in the control group, associated with a concomitant increase in leptin, may also contribute to carbohydrate metabolism disorders occurring in the course of PCOS.

## Introduction

Polycystic ovary syndrome (PCOS) is one of the most common endocrinopathies in women of childbearing age, as well as the leading cause of infertility, with the highest occurrence rate between age 20 and 30 years.^1^ The etiology of PCOS is still poorly understood. Currently, two concepts are most often invoked: the neuroendocrine origin theory of PCOS and the epigenetic theory. The first concept indicates that disturbances in the functioning of the pituitary–hypothalamus axis, leading to hyperandrogenism, play a key role.^2^ The latter concept presents that processes such as histone acetylation, DNA methylation, and post-transcriptional modification of proteins are crucial in the etiology of PCOS. In recent years, a number of mutations related to the etiopathogenesis of PCOS have been identified.^3^

PCOS is a chronic disease with menstrual disorders occurring in 93% of women.^1,4–5^ Clinical hyperandrogenism manifests mainly in the form of hirsutism, which is associated with an increased number of hair follicles and greater hair thickness and pigmentation, and affects hormone-dependent zones, such as the upper lip, chin, chest, back, upper abdomen, thighs, and arms. Hirsutism is considered the most important and common manifestation of hyperandrogenism in women with PCOS, occurring in 65%–75% of cases. However, its prevalence differs depending on race and ethnicity.^1^

PCOS has three phenotypes, namely, metabolic, hyperandrogenic, and reproductive.^1^ The most common phenotype is metabolic: obesity affects 30%–60% of the patients, often accompanied with carbohydrate metabolism disorders, such as impaired glucose tolerance (31%–35%) and type 2 diabetes (7.5%–10.0%).^6^ Central obesity appears to play a major role in the pathogenesis of metabolic disorders occurring in the course of PCOS. Insulin resistance and carbohydrate disorders are associated with dysfunctional secretion of various adipokines by adipose tissue, in patients with PCOS with and without obesity.^5^

Leptin, discovered in 1994, is a protein encoded by the *ob* gene on chromosome 7. It is biosynthesized by the adipose tissue, within the gastric and breast epithelial cells, skeletal muscles, brain, stellate cells in the liver, and placenta. This hormone plays a role in the regulation of appetite and influences glucose metabolism, lipogenesis, lipolysis, thermogenesis, and functions of macrophages and T lymphocytes.^7–8^ Previous studies have shown that in individuals with obesity, the mechanism of leptin action is disrupted–despite the presence of a high concentration of this hormone in the circulation, appetite is not suppressed and thus leptin resistance develops.^9–10^ Leptin resistance is often associated with hyperinsulinemia and insulin resistance, which may lead to disturbances in energy homeostasis and development of metabolic diseases.^10–11^

Apelin was first isolated in 1998. The most common isoform consists of 36 amino acids. Apelin is encoded by the gene APLN, located on the X chromosome. Apelin isoforms differ in biological activity depending on the length of the polypeptide chain.^12^ The most biologically active isoform of apelin is apelin-13, which is present in the mammary gland and hypothalamus. Moreover, the most common isoform (apelin-36) is located in the lungs, uterus, and testicles.^13^ Studies have documented a significant role of apelin in the course of metabolic diseases, regulation of the appetite center function, adipose tissue growth, and pathogenesis of obesity.^14,15^ Apelin reduces blood glucose, increasing its uptake and absorption by muscle cells and adipose tissue. It also increases the sensitivity of cells to insulin. Apelin, through the apelin receptor, affects the glucose transporter type 4 (GLUT4), thus stimulating glucose uptake.^16^

Visfatin is a product of the growth factor gene for early B cells, identified in 2005 in visceral adipose tissue.^17^ At present, visfatin is widely known to be produced by hepatocytes, myocytes, and lymphocytes.^18^ Key functions of visfatin include induction of preadipocyte differentiation into adipocytes and stimulation of the production of cytokines such as tumor necrosis factor alpha, interleukin-1beta, and interleukin-6.

Despite the great interest in the field of endocrinopathies among women of childbearing age, the exact mechanism of metabolic abnormalities in PCOS has not been completely understood. Studies have reported a potential link between altered type of adipokines produced by adipose tissue and metabolic disorders characterizing PCOS.^19–21^ It has been postulated that leptin regulates energy, lipid, and carbohydrate metabolisms and potentially may modulate the clinical course of PCOS. According to Altinkaya et al., apelin can serve as a specific biomarker of insulin sensitivity and lipid profile^22^ and may play a potential role in follicular growth arrest and ovulatory dysfunction.^14^

Therefore, this study aimed to evaluate adipokines, leptin, apelin, and visfatin, with regard to carbohydrate metabolism parameters, in patients diagnosed *de novo* with PCOS.

## Research Material And Methods

### Patient characteristics and study design

The study was conducted in Bydgoszcz, Poland, in two consecutive years (2013–2014). The study group consisted of 40 women, who were patients of the Department of Obstetrics, Women's Diseases and Gynecological Oncology, Collegium Medicum in Bydgoszcz, Copernicus University, Torun′. All patients had been diagnosed *de novo* with PCOS in accordance with the European Society of Human Reproduction and Embryology/American Society for Reproductive Medicine criteria from 2003.^23^ The inclusion criteria were as follows: 1) age >18 years and 2) at least two of three features (oligo- or amenorrhea, clinical and/or biochemical signs of hyperandrogenism, and ultrasonographic appearance of a polycystic ovary). Patients with other endocrinopathies such as hormonally active adrenal tumors, hyperprolactinemia, Cushing syndrome, hypothyroidism, and prolactinoma were excluded.

The control group consisted of 37 clinically healthy age-matched women presenting to the Clinical Gynecological Outpatient Clinic of the University Hospital No. 2 in Bydgoszcz for routine annual preventive visit. All controls had regular menses and had no clinical or biochemical signs of hyperandrogenism.

All participants from both the study group and control group underwent detailed physical examination. Participants’ age, height, and weight were estimated. Body mass index (BMI) was calculated as weight divided by the square of height (kg/m^2^). The Ferriman–Gallwey scale was used to assess the severity of hair presence from 0 to 4 in nine body regions (hirsutism).

The study was approved by the Bioethical Committee of Copernicus University, Torun′, Ludwik Rydygier Collegium Medicum, Bydgoszcz (No. 537/2012). Written informed consent was obtained from all participants.

### Sample preparation and measurements

Blood samples were obtained in the morning (between 7:30 and 9:00), after an overnight fasting (12 h), from an antecubital vein and placed in plastic tubes containing 1) 3.2% sodium citrate (BD Vacutainer®, Becton Dickinson, Franklin Lakes, NJ, USA) and 2) clotting activator (BD Vacutainer®, Becton Dickinson). All samples were centrifuged at 2500 × g for 20 min, and the resulting plasma and serum were stored at − 80°C until analysis but no later than 6 months.

### Laboratory measurements

The concentrations of leptin [Human Leptin ELISA (RD191001100; BioVendor Laboratory Medicine Inc., Czech Republic)], apelin (RayBio Human Apelin C Test–Terminus Enzyme Immunoassay Kit (EIA-APC); RayBiotech Inc., GA, USA), visfatin (RayBio Human Visfatin Enzyme Immunoassay Kit (EIA-VIS-1); RayBiotech Inc.), and insulin (Insulin ELISA Test (ME E-0900); LDN Labor Diagnostika Nord Gmbh & Co. KG, Germany) were determined by the enzyme-linked immunosorbent assay (ELISA).

Glucose concentration was determined by a spectrophotometric method (Glucose Colorimetric/Fluorometric Assay Kit (K606-100); BioVision Inc., CA, USA). Homeostatic model assessment for insulin resistance (HOMA-IR) =  [insulin (μU/ml) ×  glucose (mg/dl)]/405 was calculated for the assessment of insulin sensitivity. The immunoreactive insulin (IRI)/glucose index was also determined.

Leptin, apelin, visfatin, insulin, and glucose concentrations were determined at the Department of Pathophysiology, Collegium Medicum, Bydgoszcz, Copernicus University, Torun′.

### Statistical analysis

All statistical analyses were performed using Statistica version 13.3 for Windows (Statsoft, Poland). The sample size was calculated using power and sample size calculation program (at a power of 80% and a two-tailed significance level of < 0.05) (Statsoft, Poland). The Shapiro–Wilk test was applied to assess the normality of data distribution. To compare proportions of patients between groups, chi-square test was used. Data were distributed nonnormally, and the Mann–Whitney rank-sum test was used, and variables were expressed as median and interquartile range (IQR). The correlations were sought by Spearmann's rank method; *p* values <  0.05 were considered significant.

## Results

The clinical characteristics of women from the study group and control group are presented in Table 1.

As shown in Table 1, no significant differences were found in age, weight, and BMI between the PCOS group and control group. The Ferriman–Gallwey scores as well as proportions of women with menstrual disturbances and skin changes were significantly higher in the PCOS group than in the control group.

Table 2 presents the values of the tested parameters in the PCOS group and control group. Analyses revealed significantly higher values of leptin, insulin, HOMA-IR index, and IRI/glucose index in the PCOS group than in the control group. This study also shows that the concentration of apelin was >5 times lower in the PCOS group than in the control group.

In the PCOS group, a moderate positive correlation was observed between the concentrations of insulin and leptin (r = 0.6292; p < 0.0001) and concentrations of leptin and IRI/glucose (r = 0.4311; p = 0.0097). A similar finding was observed in the control group (respectively, r = 0.5344; p = 0.0009; r = 0.4311; p = 0.0097). Data from the PCOS group were subdivided based on BMI values, with a threshold of 25 (Table 3). Significantly higher concentrations of leptin, insulin, HOMA-IR index, and IRI/glucose index were found in patients of the PCOS group with BMI ≥  25 than in those with normal BMI.

In the PCOS group, a moderate positive correlation was found between BMI and leptin concentrations (r = 0.7176; p < 0.0001) and carbohydrate metabolism parameters, such as insulin (r = 0.5524; p = 0.0003), glucose (r = 0.3843; p = 0.0157), HOMA-IR index (r = 0.5895; p = < 0.0001) and IRI/glucose (r = 0.3872; p = 0.0163). A similar phenomenon was observed in the control group. Among patients with PCOS, the concentrations of selected adipokines and carbohydrate metabolism parameters were analyzed depending on the HOMA-IR value. Women with HOMA-IR ≥  2.5 had significantly higher concentrations of leptin (11.88 ng/ml vs 7.46 ng /ml; p = 0.0154), insulin (18.12 μIU/ml vs 12.44 μIU/ml; p = 0.0001), and glucose (85.60 mg/dl vs 67.20 mg/dl; p = 0.0001). A significant moderate correlation was found between HOMA-IR and leptin concentration (r = 0.6064; p < 0.0001).

## Discussion

In this study, the concentrations of adipokines, leptin, apelin, and visfatin were assessed in patients diagnosed *de novo* with PCOS with regard to selected parameters of carbohydrate metabolism.

The concentration of leptin was significantly higher in the PCOS group than in the control group (9.00 ng/ml vs 5.59 ng/ml). Moreover, leptin concentration was nearly threefold higher in women with BMI >25 kg/m^2^ than in women with normal BMI. Furthermore, the concentration of leptin correlated with the concentrations of insulin, glucose, HOMA-IR index, and IRI/glucose. Moreover, such correlations were not observed within the control group.

These results are consistent with other studies, including that of Chakrabarti in 2013,^19^ Jalilian et al., in 2016,^20^ and Mohaisen et al., in 2019.^21^ Chakrabarti found positive correlations between leptin levels and both BMI and insulin in 16 women with PCOS. Jalilian et al., also found increased leptin levels in women with PCOS having obesity compared with women with lean body frame. However, they found no correlation between leptin and insulin levels. Jahromi et al., also found significant association of leptin with BMI as well as of leptin with carbohydrate metabolism indices.^24^

Previous studies have shown that in individuals with obesity, the mechanism of action of leptin is disrupted–despite the presence of a high concentration of this hormone in the circulation, appetite is not suppressed and thus leptin resistance develops.^9–10^ Leptin resistance is often associated with hyperinsulinemia and insulin resistance, which may lead to disturbances in energy homeostasis and development of numerous metabolic diseases.^10–11^ These observations became the basis for determining the HOMA-IR index in the PCOS group, which is one of the most frequently used methods of indirect assessment of insulin sensitivity.

In this study, the study group was divided into two subgroups with HOMA-IR ≥ 2.5 and HOMA-IR < .5. Among women with HOMA-IR ≥ 2.5, the leptin concentration was significantly higher. The obtained results are consistent with those of studies, in which increasing insulin resistance was observed among women with PCOS having obesity.^3,25–27^

Interestingly, this study reveals a significantly lower level of apelin in the PCOS group than in the control group. In the PCOS group, the concentration of apelin was >5 times lower and was independent of BMI and carbohydrate metabolism parameters. However, studies on apelin have varied and inconclusive results.^22,28–30^ Altinkaya et al., also found that women with PCOS exhibited lower serum concentrations of apelin than controls. However, they noticed significant positive relation between apelin and BMI and HOMA-IR index.^22^ Olszanecka-Glinianowicz et al., also found lower isoforms of apelin (apelin-36 and aplein-12) in women with PCOS.^30^

The results of this study may indicate the predictive value of apelin in the context of metabolic disorders occurring in the course of PCOS, including carbohydrate metabolism. Research on apelin is worth further analysis, as is the verification of the hypothesis on whether adipokine–with significantly lower concentration in patients with PCOS–may be helpful in predicting metabolic disorders in the course of PCOS.

As regards visfatin, its concentrations were slightly higher in women with PCOS, but differences were not significant. No difference was found in visfatin levels in subgroups of women with PCOS divided according to BMI or HOMA-IR. Moreover, no significant correlations were found between visfatin and carbohydrate metabolism indices.

Visfatin also stimulates the accumulation of triglycerides in adipocytes, increasing the expression of fatty acid synthase and adiponectin, and activates the phosphorylation of insulin receptor as well as insulin receptor substrates 1 and 2^17^ This protein features insulin-mimetic properties, and by binding to the insulin receptor, it leads to an increased glucose uptake. The production of visfatin is a likely compensatory response to insulin resistance to maintain normoglycemia.^31–36^ However, in this study, visfatin values in the PCOS group did not correlate with other adipokines, as well as with carbohydrate metabolism parameters and BMI. We speculate that visfatin may play a role in severe metabolic disorders, rather than in early stages of glucose dysregulation.

This study has some limitations. First, the study group was relatively small, and the group of patients with obesity was included on the basis of their BMI–an indicator which features a number of inaccuracies. Second, the exact role of adipokines in the pathogenesis of PCOS is still debated. More detailed analysis including a comprehensive panel of adipokines should be considered for future studies.

This study shows significant changes in the concentrations of leptin and apelin. Significantly elevated levels of leptin were accompanied by changes in carbohydrate metabolism toward reduced insulin sensitivity, depending on BMI.

## Conclusions

Increased leptin concentration observed in women diagnosed *de novo* with polycystic ovary syndrome, as well as positive correlations between leptin and HOMA-IR, IRI/glucose, and BMI, may indicate a potential role of leptin in the reduction of tissue sensitivity to insulin.

Significantly lower apelin concentration in women with PCOS (>5 fold) than in women without PCOS, associated with a concomitant increase in leptin, may also contribute to carbohydrate metabolism disorders occurring in the course of PCOS.

### Conflict Of Interest

The authors stated that there are no conflicts of interest regarding the publication of this article.

## Figures and Tables

**Table 1:** Characteristics of patients diagnosed *de novo* with PCOS and healthy controls

Parameters	Group of patients with PCOS (N = 40)		Control group (N = 37)		p

	Median	IQT	Median	IQT	

Age [years]	29	26; 32	26	24; 29	ns

Weight [kg]	63.5	57.0; 70.0	59.0	55.0; 61.0	0.048

BMI	22.1	20.7; 26.1	20.9	19.6; 22.6	ns

Ferriman–Gallwey score	2.5	0.0, 7.5	0	0, 0	>0.05

		N [%]		N [%]

Menstrual disturbances	Yes	27 [67.5]	Yes	-	>0.05

	No	13 [22.5]	No	37 [100]	

Skin changes with seborrhea	Yes	17 [42.5]	Yes	6 [16,2]	>0.05

	No	23 [57.5]	No	31 [83,8]	


BMI, body mass index; IQR, interquartile range; ns, not significant; PCOS, polycystic ovary syndrome

**Table 2:** Selected adipokines and parameters of carbohydrate metabolism between the PCOS group and control group.

Parameters	PCOS group (N = 40)		Control group (N = 37)		p

	Median	IQT	Median	IQT	

Leptin [ng/ml]	9.00	5.06; 13.60	5.59	2.98; 10.75	>0.05

Apelin [ng/ml]	107.44	56.94; 179.53	608.77	109.20; 1071.03	>0.001

Visfatin [ng/ml]	24.58	18.82; 34.78	19.8	16.63; 28.34	ns

Insulin [μIU/ml]	14.37	12.08; 18.27	11.28	8.53; 14, 34	>0.001

Glucose [mg/dl]	77.80 ± 16.00	52.70–108.00	73.30 ± 14.40	44.40–101.20	ns

HOMA-IR	2.69	2.10; 3.75	1.93	1.44; 2.68	>0.001

IRI/Glucose	0.19	0.16; 0.25	0.16	0.12; 0.20	>0.01


HOMA-IR, homeostatic model assessment for insulin resistance; IRI, immunoreactive insulin; IQR, interquartile range; ns, not significant; PCOS, polycystic ovary syndrome

**Table 3:** Comparison of adipokines and carbohydrate metabolism parameters between the PCOS groups according to the body mass index

Parameters	PCOS group with BMI < 25 (N = 28)		PCOS group with BMI ≥ 25 (N = 12)		p

	Median	IQT	Median	IQT	

Leptin [ng/ml]	7.08	4.47; 9.99	20.20	10.90; 26.63	>0.001

Apelin [ng/ml ]	110.04	53.48; 237.48	103.67	58.16; 147.97	ns

Visfatin [ng/ml]	26.11	20.53; 32.38	21.33	14.43; 41.13	ns

Insulin [μIU/ml]	12.83	11.1; 16.58	20.22	15.66; 29.98	>0.001

Glucose [mg/dl]	87.15	78.25; 99.00	89.20	74.20; 94.00	ns

HOMA-IR	2.23	1.98; 3.05	3.84	3.00; 6.48	>0.001

IRI/Glucose	0.19	0.15; 0.21	0.28	0.22; 0.35	>0.01


BMI, body mass index; HOMA-IR, homeostatic model assessment for insulin resistance; IRI, immunoreactive insulin; IQT, interquartile range; ns, not significant; PCOS, polycystic ovary syndrome
